# The Combined Effect of Dietary and Behavioral Risk Factors in Gastric Cancer: A Case-Control Study Using a Healthy Lifestyle Index in Fujian, China

**DOI:** 10.3390/nu18091343

**Published:** 2026-04-24

**Authors:** Monica Wangari, Xinyu Chen, Qingying Wang, Fengqin Zou, Yaqing Wu, Yulan Lin

**Affiliations:** Department of Epidemiology and Health Statistics, Fujian Provincial Key Laboratory of Environmental Factors and Cancer, School of Public Health, Fujian Medical University, Fuzhou 350122, China

**Keywords:** gastric cancer, dietary habits, physical activity, smoking, alcohol consumption, healthy lifestyle index

## Abstract

**Background/Objectives**: Gastric cancer (GC) remains a major health challenge in high-incidence regions like Fujian Province, China. This study aimed to identify modifiable dietary and behavioral risk factors for GC and to evaluate their combined effect using a Healthy Lifestyle Index (HLI) in a high-incidence region of China. **Methods**: A case–control study was conducted at a tertiary hospital from June 2023 to December 2024, including 336 newly diagnosed GC cases and 336 healthy controls from Fujian Province. A Healthy Lifestyle Index (HLI, scored 0–10) was constructed from ten dietary and behavioral factors, with participants categorized into tertiles as healthy, moderate, or unhealthy. **Results**: The proportions of males and females were 56.5% and 43.5%, respectively. The mean age of the case group was 56.76 ± 10.83 years, significantly higher than that of the control group (53.86 ± 11.13 years, *p* < 0.001). The HLI incorporated ten behavioral/dietary components: sedentary behavior, smoking, alcohol consumption, tea drinking, physical activity, pickled and processed food intake, regular eating habits, fruit intake, vegetable intake, and red meat intake. Cases showed a higher proportion of unhealthy lifestyle patterns (34.8% vs. 26.8%), whereas controls showed a higher proportion of healthy lifestyle behaviors (41.7% vs. 37.5%); however, the overall between-group distribution of HLI categories was not statistically significant (*p* = 0.078). The multivariate logistic regression showed that the healthy group was associated with a lower risk of developing GC compared to the unhealthy group (aOR = 0.34, 95% CI: 0.20–0.57, *p* < 0.001). **Conclusions**: A healthier combined dietary and behavioral profile may be associated with lower odds of GC in this population. However, the observed associations should be interpreted cautiously because of the case–control design, the lack of *H. pylori* data, and the absence of formal validation of the HLI in the Fujian population. Prospective studies with objective dietary assessment and more detailed clinical characterization are warranted.

## 1. Introduction

Each year, more than a million new cases of GC are diagnosed worldwide, highlighting its continued significance as a global health problem. It remains the fifth most diagnosed cancer globally, despite a decline in incidence rates over the last five decades [1]. The distribution of GC cases worldwide is disproportionate. Asia has the highest burden, with 73% of all diagnoses [2]. Of the 73% of new cases in Asia, China represents half (50%). According to the GLOBOCAN International Agency for Research on Cancer 2022, GC is the fifth most diagnosed cancer in China. There is a higher incidence rate among males in China, and it increases with age. The incidence rate among males is 9.7% and 4.9% among females. Incidence rates in China are higher in rural populations than in urban populations [2,3,4].

GC has two primary anatomical forms based on tumor site: cardia (proximal) and non-cardia (distal). They are associated with distinct risk factors. Modifiable risk factors, such as obesity and reflux, are linked to cardia cancer, while *Helicobacter pylori* infection is linked to non-cardia cancer [5]. Globally, China accounts for approximately 70% of new cardia GC cases and 50% of new non-cardia GC cases [6]. Beyond anatomical classification, gastric adenocarcinoma is further distinguished histologically by the Lauren classification into two major subtypes: the intestinal and diffuse types. The intestinal type is characterized by well-differentiated, gland-forming cells, is more commonly associated with environmental and dietary risk factors such as *H. pylori* infection, high salt intake, and low fruit and vegetable consumption, and tends to predominate in older males in high-incidence regions. The diffuse type, by contrast, consists of poorly cohesive cells, is more aggressive, carries a higher propensity for peritoneal metastasis, and is less responsive to standard chemotherapy regimens. Prognostically, the intestinal type is associated with a more favorable outcome, with studies reporting a 5-year overall survival of approximately 57.7% compared with 45.6% for the diffuse type [7,8].

The development of GC involves multiple factors, with modifiable and non-modifiable risk factors such as *Helicobacter pylori* infection often interacting to cause the disease. Although *H. pylori* alone might be insufficient to cause GC, it may foster an environment in which carcinogens can interact more effectively with other risk factors [9]. Smoking has been found to have a damaging effect on the gastrointestinal tract, elevating the risk of peptic ulcers and inflammatory conditions, and increasing susceptibility to *H. pylori* infection [10]. The role of alcohol consumption is still controversial and is likely multifactorial [11]. One plausible biological relationship between alcohol consumption and GC is through ethanol, which causes damage to the gastric mucosa [12].

Regular physical activity may help prevent GC through multiple biological mechanisms. Ma et al.’s multiregional meta-analysis revealed significant protective associations between physical activity and GC outcomes, demonstrating a 17% lower incidence rate and a 24% reduction in mortality risk [13]. Diet-related exposures that have been established to increase GC risk include high-salt intake, high-fat intake, animal products, pickled vegetables, and salt-preserved foods such as fish and meat [14]. High salt levels in the stomach compromise the mucosal barrier, resulting in inflammatory responses and pathological changes, such as widespread erosion and cellular degeneration [15]. It has also been reported to further facilitate *H. pylori* colonization by altering mucin distribution across gastric mucosal cells. At the molecular level, elevated salt intake may also upregulate CagA expression and enhance its translocation into gastric epithelial cells, amplifying *H. pylori*’s capacity to disrupt normal gastric function [16]. *H. pylori* drives carcinogenesis through chronic inflammation, DNA damage, oxidative stress, and the action of virulence factors such as CagA and VacA, which together promote genomic instability and malignant transformation of the gastric epithelium [17]. Antioxidant-rich foods, particularly fruits and vegetables, may partially mitigate *H. pylori*’s carcinogenic effect by scavenging reactive radical species, inducing detoxification enzymes, and interfering with the formation of carcinogenic N-nitroso compounds in the gastric mucosa. Conversely, fruits, vegetables, whole grains, and nuts have been observed to have a protective effect against GC [18,19].

Despite the outstanding achievements in reducing incidence and mortality rates in China, nearly half of the new GC cases remain attributable to modifiable risk factors [6]. This high incidence carries a significant economic and financial burden at the individual patient, family/caregiver, and national levels. In 2017, the total expense of cancer was 304.8 billion Chinese Yuan (CNY). The inpatient expense was 241.9 CNY, with GC accounting for the top three inpatient cases [20]. A study by Zhang et al. reported that patients spent 63.8% of their annual household income solely on treatment, and that 79.2% of these families found this cost highly unmanageable. The time loss due to travel and out-of-work days seeking treatment also had an indirect economic impact [21].

This has prompted more studies to keenly examine established modifiable risk factors, their role in elevating GC risk, and how they can be used as interventions to prevent GC [22]. Although extensive research has pinpointed multiple independent risk factors for GC, there remains a significant knowledge gap regarding their combined effects. Current literature lacks a comprehensive investigation into how different levels of exposure, consumption patterns, and interactions between known risk factors may work together to either increase or decrease the likelihood of GC development [23,24]. Evidence regarding the interplay between behavioral risk factors (smoking, alcohol consumption, and physical inactivity) and dietary habits is considerably limited.

To address these knowledge gaps, this research aimed to examine the lifestyle factors that contribute to GC risk. We hypothesized that a greater cumulative burden of unhealthy dietary and behavioral factors, as captured by the HLI, would be significantly and independently associated with increased GC risk, even after adjustment for demographic confounders. Subsequently, we focused on understanding how the combination of these factors, both risk and protective factors, works towards the risk of GC.

## 2. Materials and Methods

### 2.1. Study Design and Study Participants

A case–control study was conducted between June 2023 and December 2024 at the Union Hospital of Fujian Medical University, China. Age matching was attempted but was not feasible due to substantial age differences between the case and control populations. All participants were living in different districts and counties of Fujian province and met the inclusion and exclusion criteria. The patients or their family members signed consent forms for this study.

Patients clinically diagnosed with primary GC were recruited as cases. Our study enrolled histopathologically confirmed primary GC cases without restriction to subtype. The inclusion criteria for the case group were: (1) Age ≥ 18 years; (2) Newly diagnosed cases of GC confirmed by histopathology or cytology; (3) Aware of their diagnosis; (4) Understand the purpose of the study and voluntarily agree to participate. The exclusion criteria were: (1) History of cancer; (2) Patients with severe language communication or cognitive impairment.

The control group consisted of healthy residents who had never been diagnosed with GC. The inclusion criteria were: (1) Age ≥ 18 years; (2) Long-term residents of Fujian Province (those who have lived in the survey area for at least 6 months within the 12 months prior to the survey); (3) Able to communicate effectively; (4) Understand the purpose of the study and voluntarily agree to participate. The exclusion criteria were: (1) History of cancer; (2) History of severe chronic disease, e.g., diabetes, chronic liver disease, kidney disease; (3) Patients with severe language communication impairment or cognitive impairment. Individuals with a history of severe chronic disease were excluded because such conditions may independently influence dietary habits and lifestyle behaviors, thereby introducing confounding.

### 2.2. Data Collection

Trained interviewers conducted face-to-face surveys among study participants, using a standardized epidemiological questionnaire developed by our research group. The questionnaire included sections on (I.) General information, (II.) Behavioral lifestyle, (III.) Dietary habits, (IV.) Personal and family health, (V.) Mental, sleep, and emotional status, (VI.) Physical activity, (VII.) Female reproductive health, and (VIII.) Food frequency questionnaire. The dietary section of this questionnaire, including the 78-item semi-quantitative FFQ, had been previously validated in adults from Fujian, China, using a 1-month test–retest design and three-day 24 h dietary recalls as the reference method [25]. The FFQ showed moderate-to-good reliability and validity, with reliability correlations of 0.60–0.80 for food groups and 0.66–0.96 for energy/nutrients, relative validity correlations of 0.41–0.72 for food groups and 0.40–0.70 for nutrients, and 78.8–95.1% of participants classified into the same or adjacent tertile.

#### 2.2.1. General Demographic Information

The data collected by the general demographic questionnaire mainly included: name, gender, age, height, weight, ethnicity, place of birth, marital status, education level, occupation, average monthly family income, type of medical insurance, history of surgery or chemotherapy, and daily life stress level. Body mass index (BMI) was calculated based on reported height and weight.

#### 2.2.2. Smoking and Alcohol Consumption

Smokers were defined as those who smoked at least one cigarette daily for over six consecutive months, or who had smoked a total of 150 or more cigarettes. Those who consumed alcohol at least once a week for more than six months were classified as drinkers.

#### 2.2.3. Tea Drinking

Tea drinking was categorized as “Low” and “High”, based on the survey question, “How often did you drink tea in the past year?”. Those who responded 5 = Quit, 0 = Hardly ever (<1 time/month), and 1 = Occasionally (1–3 times/month) were classified as Low, while 2 = Rarely (1–2 days/week), 3 = Often (3–5 days/week), and 4 = Almost every day were High.

#### 2.2.4. Physical Activity

The questionnaire related to physical activity included three parts: Urban Residents, Rural Residents, and a Common part. Physical activity was grouped into occupational, commuting, leisure, and domestic physical activities. Time spent on moderate-to-vigorous physical activity (MVPA) per week was calculated. These were activities that had Metabolic Equivalent Tasks (METs) greater than 3 (Table S1). MET scores for all physical activities were directly extracted from the 2024 Adult Compendium of Physical Activities [26].

#### 2.2.5. Sedentary Behavior

Self-reported sedentary time was assessed by asking participants to estimate their average daily hours engaged in sedentary behaviors, including sitting, watching television, and other screen-based activities. Sedentary behavior was categorized as Low (<4 h/day) or High (≥4 h/day) based on established thresholds used in prior literature [27].

#### 2.2.6. Dietary Habits

Regular eating habits were assessed using the question, “Do you have meals regularly?” with responses “Yes” and “No”.

#### 2.2.7. Food Frequency Questionnaire (FFQ)

The FFQ included 78 individual food items, and the food frequency range of the FFQ was: ① = ≥4 times a day; ② = 2–3 times a day; ③ = once a day; ④ = 4–6 times a week; ⑤ = 2–3 times a week; ⑥ = once a week; ⑦ = 1–3 times a month; ⑧ = occasionally; ⑨ = do not eat.

### 2.3. Construction of the Healthy Lifestyle Index Score

To examine the combined effects of lifestyle factors on GC risk, we created a Healthy Lifestyle Index (HLI) based on an approach from previous studies [28,29]. The index included 10 components: fruit, red meat, and vegetable intake; pickled and processed food consumption; smoking status and alcohol consumption; physical activity and sedentary behavior; tea drinking; and regular eating habits. Healthy behaviors received 0 points and unhealthy behaviors received 1 point (Table S2). The total Healthy Lifestyle Index score was calculated by summing the scores for all components included. For analytical purposes, the total HLI scores (0–10) were divided into tertiles to represent three distinct lifestyle patterns: low-risk (healthy), moderate-risk (moderate), and high-risk (unhealthy) groups. For all food components, frequencies were first converted to daily intake, then multiplied by standard serving sizes. Standard servings were assigned to each food item based on commonly consumed portions by the Chinese population [30]. Food intake levels of relevant components in our study were then compared to the Dietary Guidelines for Chinese Residents 2022 [31]. Recommended intake levels for our specific food components were ≥200–350 g/day for fruits, ≥300–500 g/day for vegetables, ≤5 g/day for pickled and processed meat, and ≤300–500 g/week or twice per week for red meat [32]. Participants who met the recommended intake were classified as healthy (0 points), while those who did not were classified as unhealthy (1 point), as shown in Table S2.

Alcohol consumption and smoking status were categorized using the thresholds defined in Section 2.2.2, and sedentary behavior used the threshold in Section 2.2.5. Physical activity assessment was based on the physical activity guidelines for the Chinese population [33]. Participants meeting the recommended ≥150 min per week were classified as healthy (0 points), while those with <150 min per week were classified as unhealthy (1 point). Tea drinking was categorized as healthy (0 points) for daily consumption, while less than once per month, 1–3 times per month, 1–2 days per week, or quitting were considered unhealthy (1 point). There was no standard guideline for tea drinking, but a study by He and Lyu found that daily tea consumption was associated with health benefits [34]. Based on existing literature, the presence of irregular eating patterns was classified as unhealthy (1 point), while the absence of the habit was classified as healthy (0 points) [35].

### 2.4. Statistical Analysis

Descriptive statistics (means, frequencies) were computed to characterize baseline demographics. A chi-square test of independence (χ^2^ test) was performed to assess statistically significant associations between demographic and behavioral factors and GC status. Continuous variables were compared using the independent-samples *t*-test.

Univariate binary logistic regression was then used to estimate odds ratios (ORs), 95% confidence intervals (CIs), and *p* values for all demographic characteristics and independent variables. Multivariable logistic regression models were constructed to assess associations with GC, with covariates selected a priori based on prior literature and biological plausibility rather than on univariate statistical significance alone. The adjusted models included age, sex, marital status, education level, occupation, average monthly household income, and residence. Adjusted ORs, 95% CIs, and *p* values were obtained. For ease of interpretation, odds ratios are presented as percentage changes: for ORs > 1, percentage increase = (OR − 1) × 100%; for ORs < 1, percentage decrease = (1 − OR) × 100%.

To examine the combined effect of lifestyle factors, the Healthy Lifestyle Index (HLI), categorized as Healthy, Moderate, and Unhealthy, was assessed in both univariate and adjusted logistic regression models. The model was adjusted for age (continuous), sex, marital status, income, educational attainment, occupation, and residence. Model calibration was verified using the Hosmer–Lemeshow goodness-of-fit test, and discriminative ability was assessed via the Area Under the ROC Curve (AUC). To evaluate potential synergistic effects between lifestyle behaviors, multiplicative interaction terms for biologically plausible pairs were entered into the model, with statistical significance set at *p* < 0.05. Independence of HLI components was confirmed via Spearman’s rank correlation across all ten binary items, and multicollinearity was assessed using the Variance Inflation Factor (VIF) analysis. To assess the robustness of findings, two sensitivity analyses were conducted: first, the HLI was modeled as a continuous variable to evaluate dose–response associations; second, sex-stratified analyses were performed to examine whether associations differed between males and females. All tests were two-sided, with significance set at *p* < 0.05. Analyses were performed using IBM SPSS Statistics, version 26.0 (IBM Corp., Armonk, NY, USA) [36].

## 3. Results

### 3.1. Baseline Demographics

Table 1 presents the baseline characteristics of the 672 participants (336 cases, 336 controls). Cases were significantly older than controls (56.76 ± 10.34 vs. 53.86 ± 11.13 years, *p* < 0.001), with a greater proportion aged over 55 (60.7% vs. 44.0%). Sex was evenly distributed by design (56.5% male in both groups). Cases were more likely to be married (93.8% vs. 88.1%, *p* = 0.011), have lower educational attainment (*p* = 0.047), and work in non-professional occupations (*p* = 0.012). Income and BMI did not differ significantly between groups.

Regarding lifestyle factors, smoking was more prevalent among cases (39.0% vs. 30.4%, *p* = 0.019), as was high sedentary behavior (52.7% vs. 14.3%, *p* < 0.001). Cases were more likely to consume pickled foods in excess of recommended levels (31.0% vs. 11.3%, *p* < 0.001) and report irregular eating habits (72.3% vs. 45.2%, *p* < 0.001). Daily tea drinking was less common among cases (19.6% vs. 11.0%, *p* = 0.002). No significant differences were observed for fruit, red meat, or vegetable intake. Alcohol consumption did not differ between groups (*p* = 0.916).

### 3.2. Association Between Individual Lifestyle Factors and GC Risk

In univariate logistic regression, several behavioral factors were significantly associated with GC, as shown in Table 2. High sedentary behavior was strongly associated with increased odds (OR = 3.11, 95% CI: 2.09–4.63, *p* < 0.001), as was insufficient physical activity, where those falling below the recommended 150 min per week of moderate-to-vigorous activity had more than double the odds compared to those meeting the guideline (OR = 2.46, 95% CI: 1.68–3.59, *p* < 0.001). Irregular meal habits similarly showed a substantial association with increased risk (OR = 3.08, 95% CI: 2.15–4.43, *p* < 0.001). High pickled and processed food consumption also demonstrated a notably elevated crude association (OR = 3.52, 95% CI: 2.33–5.29, *p* < 0.001), suggesting a strong unadjusted relationship with GC risk. Less than daily tea drinking showed a modest but significant association (OR = 1.98, 95% CI: 1.25–3.05, *p* = 0.002), while smoking, alcohol, fruit, red meat, and vegetable intake did not reach statistical significance at the univariate level.

After adjustment in the multivariable model, sedentary behavior remained the strongest behavioral risk factor (adjusted OR = 4.06, 95% CI: 2.59–6.37, *p* < 0.001), with its effect actually increasing. Insufficient physical activity similarly remained independently significant (adjusted OR = 1.98, 95% CI: 1.31–2.99, *p* < 0.001), and notably, both behaviors retained significance simultaneously, reinforcing the idea that prolonged inactivity and insufficient exercise represent distinct and compounding risks. Irregular meal habits also maintained a significant independent association (adjusted OR = 2.11, 95% CI: 1.47–3.03, *p* < 0.001). Pickled food consumption, while attenuated, similarly remained significant after adjustment (adjusted OR = 1.75, 95% CI: 1.06–2.89, *p* = 0.030). Tea drinking, by contrast, lost significance in the multivariable model (adjusted OR = 1.34, 95% CI: 0.82–2.20, *p* = 0.244), suggesting that its apparent univariate association was largely confounded by other behavioral factors rather than reflecting an independent effect.

### 3.3. Association Between the Healthy Lifestyle Index and GC Risk

Spearman rank correlations confirmed statistical independence among the ten HLI components (all ρ < 0.3), and all VIF values were below 5, indicating no multicollinearity among predictors (Tables S3 and S4).

Table 3 presents the distribution of HLI categories across cases and controls. Although the difference between groups did not reach statistical significance (*p* = 0.078), the descriptive distribution suggested a possible pattern, where cases were more likely to fall into the unhealthy lifestyle tertile (34.8% vs. 26.8%), and controls were more concentrated in the healthy tertile (41.7% vs. 37.5%). The slightly higher mean HLI among cases (4.91 ± 1.47 vs. 4.71 ± 1.35, *p* = 0.063) was directionally consistent with this pattern, although the difference was not statistically significant.

Table 4 shows the association between HLI categories and GC risk. Using the unhealthy lifestyle tertile as the reference, both moderate and healthy lifestyles were associated with significantly reduced odds of being a case. In the univariate analysis, this reduction was modest and comparable across both categories (OR 0.68 and 0.69, respectively), suggesting a possible association between healthier lifestyle patterns and lower odds of GC. However, after adjusting for confounders, the healthy lifestyle category showed a notably stronger and more meaningful reduction in odds (adjusted OR 0.34, 95% CI: 0.20–0.57, *p* < 0.001) compared to the moderate category (adjusted OR 0.56, 95% CI: 0.31–1.00, *p* = 0.049). This difference between crude and adjusted estimates indicates that the observed association was sensitive to adjustment for measured confounders. The observed gradient across HLI categories was consistent with a possible dose–response pattern; however, this finding should be interpreted cautiously in light of the observational design and potential residual confounding.

The adjusted model demonstrated good calibration (Hosmer–Lemeshow χ^2^(8) = 5.595, *p* = 0.692) and acceptable discriminative ability, as evidenced by the Area Under the Receiver Operating Characteristic Curve (AUC = 0.754, 95% CI: 0.711–0.797, *p* < 0.001), supporting the validity of the regression model. Model validity is provided in Supplementary Table S5 and Figure S1. Additionally, we tested for multiplicative interactions between key lifestyle factors (smoking × alcohol consumption, red meat × vegetable intake, and physical activity × sedentary behavior). No significant interactions were observed (all *p* > 0.05), indicating that the components of the HLI act as independent risk factors.

To further examine the consistency of these findings, two additional analyses were conducted (Table 5). When the HLI was modeled as a continuous variable, each one-unit increase in HLI score was associated with a 34% increase in the odds of GC after full adjustment (AOR = 1.34, 95% CI: 1.15–1.55, *p* < 0.001), which was consistent with a possible dose–response relationship between the accumulation of unhealthy lifestyle behaviors and GC risk.

In sex-stratified analyses, the association between HLI and GC appeared more evident among males than females. Among males, both the moderate (AOR = 0.29, 95% CI: 0.15–0.58, *p* < 0.001) and healthy (AOR = 0.35, 95% CI: 0.17–0.73, *p* = 0.005) categories were significantly protective relative to the unhealthy category. Among females, the overall HLI association remained significant (*p* = 0.013), though individual category comparisons were attenuated, with neither the moderate (AOR = 0.45, 95% CI: 0.17–1.15, *p* = 0.094) nor the healthy (AOR = 1.32, 95% CI: 0.47–3.72, *p* = 0.604) categories reaching statistical significance. This attenuation may reflect differences in lifestyle exposure patterns, such as smoking and alcohol consumption, between sexes. Collectively, these additional analyses provided some support for the consistency of the primary findings, although the results should be interpreted cautiously.

## 4. Discussion

Our study primarily aimed to assess the combined effect of behavioral risk factors on GC risk among residents of Fujian Province. By creating a Healthy Lifestyle Index, we evaluated how the accumulation of these factors influences the likelihood of GC. The Healthy Lifestyle Index comprised 10 factors: alcohol consumption, smoking, tea drinking, physical activity, sedentary behavior, vegetable intake, pickled and processed food intake, regular eating habits, fruit intake, and red meat intake. After adjustment for measured confounders, participants in the healthy HLI category had lower odds of GC than those in the unhealthy category (AOR = 0.34, 95% CI: 0.20–0.57). The overall pattern of findings was consistent with the possibility that healthier combined dietary and behavioral profiles may be associated with lower odds of GC. However, these findings should be interpreted cautiously, given the case–control design, the lack of *H. pylori* data, and the absence of formal validation of the HLI in this population.

In our adjusted model, participants in the healthiest HLI category had 66% lower odds of GC than those in the unhealthy category. This is broadly consistent with previous studies reporting that healthier lifestyle patterns are associated with lower disease risk and mortality. One such study is the 45 and Up Australian Study cohort, which examined the combined effect of smoking, alcohol use, dietary behavior, sedentary habits, physical activity and sleep on all-cause mortality, especially non-communicable diseases. They concluded that maintaining a healthy lifestyle could not only lower the risk of GC but also reduce all-cause mortality [29]. Another cohort study focusing on the combined effect of smoking, alcohol, obesity, high sodium intake, and a diet with low fruit and vegetable intake and high meat intake in GC among the Singapore Chinese population found that higher healthy composite scores were associated with reduced risk of GC. Compared to the unhealthy group, the healthiest group exhibited a 58% reduction in risk of GC, both cardia and non-cardia GC [37].

The robustness of our HLI is further supported by its performance as a continuous variable (AOR = 1.34, 95% CI: 1.15–1.55, *p* < 0.001). This result was consistent with a possible dose–response pattern, with each 1-unit increase in the HLI score (representing the addition of an unhealthy behavior) suggesting that the odds of developing GC increased by 34%. This linear trend shows that even a single unhealthy habit meaningfully contributes to a higher disease risk profile. However, our sex-stratified analysis revealed a stark contrast between sexes. In males, the protective effect of a healthy lifestyle was highly significant compared to the unhealthy reference group. Males in the healthy category saw a 65% reduction in GC odds (AOR = 0.35, 95% CI: 0.17–0.73, *p* = 0.005). In contrast, the association did not reach statistical significance among females (AOR = 1.32, 95% CI: 0.47–3.72, *p* = 0.604). It is possible that the specific behavioral factors weighted in our HLI, such as smoking, alcohol consumption, and certain dietary habits, are more dominant drivers for males in Fujian Province, whereas female GC risk might be more heavily influenced by factors not fully captured by this index, such as hormonal variations or specific environmental exposures. Although the association appeared clearer in the male and continuous analyses, the sex-stratified results should be interpreted cautiously, particularly given the limited precision of the female-specific estimates.

Several individual risk factors remained significantly associated with GC after adjustment. Participants who reported more than 4 h of daily sedentary time had significantly higher odds of GC (AOR = 4.06, 95% CI: 2.59–6.37). The relationship between sedentary behavior and cancer risk has garnered increasing attention as a distinct exposure, independent of physical inactivity. Sedentary behavior, defined as prolonged sitting or reclining and characterized by low energy expenditure, is associated with adverse cardiometabolic profiles and has been linked to an increased risk of several cancers, with proposed biological pathways including adiposity and metabolic dysfunction. The main biological mechanisms through which sedentary behavior may promote carcinogenesis include dysregulation of endogenous sex steroids and metabolic hormones, impaired insulin sensitivity, and the promotion of chronic inflammation [38,39].

Our study further showed that sedentary behavior and physical activity present distinct risk factors and should be addressed individually in the promotion of healthy lifestyle interventions. Participants who did not meet the WHO physical activity recommendations (150 min/week of moderate-to-vigorous physical activity) had significantly increased odds of GC (AOR = 1.98, 95% CI: 1.31–2.99). The effects of physical activity and general health have been widely explored, and it remains a key modifiable determinant of health. Physical activity lowers inflammation, increases insulin sensitivity, and prevents tumor metabolism [40]. Numerous studies, including a meta-analysis by Psaltopoulou et al. [41], have concluded that physical activity reduces the risk of GC, as was also indicated by our findings.

Two dietary patterns remained significant in the adjusted model. Consumption of pickled and processed foods demonstrated a significant association with GC (AOR = 1.75, 95% CI: 1.06–2.89). This association is well supported in the literature. A large meta-analysis examining 60 epidemiological studies found that the vast majority of studies reported risk estimates above one for GC among consumers of pickled vegetables or foods. The pooled odds ratio was 1.52 (95% CI: 1.37–1.68) for the overall association, with stronger effects observed in Korean and Chinese populations [42]. A subsequent dose–response meta-analysis further reinforced this evidence, demonstrating that the pooled risk of GC incidence was approximately 1.15 times higher for every 40 g/day increment in pickled vegetable intake. The biological plausibility is well established. Pickled vegetables and salted fish are exogenous sources of sodium nitrates and nitrites, which react with amino acids in the stomach to form N-nitroso compounds. Additionally, high salt concentrations may damage the mucosal barrier, promoting inflammation, erosion, and degeneration of the gastric mucosa [43]. Similarly, we found irregular eating habits to be significantly associated with GC in this population (AOR = 2.11, 95% CI: 1.47–3.03). Eating irregularities, such as skipping meals, have been associated with increased risk of GC [44]. It has especially been found to increase the risk of *Helicobacter pylori* infection, which is an established risk factor for GC [45].

Several limitations should be considered when interpreting our findings. First, the case–control design precludes establishing temporal relationships or causal inference. Second, the reliance on self-reported data for lifestyle behaviors and anthropometric measurements, such as height and weight for BMI, sedentary behavior, and dietary patterns, introduces potential recall and social desirability bias, which may have led to misclassification of exposure categories. In addition, our use of community volunteers as controls may have introduced healthy volunteer bias, as these individuals may have been more health-conscious than the general population of Fujian Province, potentially overestimating the observed risk differences. Third, although the HLI in this study was developed with reference to previously published indices and national guidelines, it has not been formally validated in the Fujian population. Future studies should further evaluate its reproducibility and validity in larger prospective datasets. Fourth, several potentially important confounders were not incorporated into the present analysis. Most notably, *H. pylori* infection status, a major risk factor for GC, was unavailable and may therefore have contributed to residual confounding. In addition, information on medication use (e.g., proton pump inhibitors) and history of gastric diseases was not included in the current analysis, which may also have biased the observed associations. Although we adjusted for age in our multivariable models, the possibility of residual confounding or age-related selection bias cannot be entirely excluded. Fifth, our analysis was limited by the absence of GC subtype data. The lack of anatomical and histological classification prevented a targeted assessment of subtype-specific risks and limited interpretation of whether dietary and behavioral associations differed by GC subtype. Finally, although we performed comprehensive adjustments, unmeasured factors and cultural specificities mean that these findings may not be fully generalizable to populations with different dietary patterns or genetic backgrounds. Future research should incorporate objective anthropometric measurements, serological testing for *H. pylori* infection status, and subtype stratification to provide a more granular understanding of lifestyle-related GC risk.

## 5. Conclusions

This study suggests that several modifiable dietary and behavioral factors may be associated with GC odds, both individually and cumulatively. Despite the well-established evidence linking lifestyle habits to GC risk, patients frequently fail to recognize these modifiable risk factors. Healthcare professionals are therefore uniquely positioned to bridge this knowledge gap through structured lifestyle counseling. Routine clinical encounters should be leveraged to educate patients, particularly those in high-risk groups, on the harmful effects of excessive salt intake, the benefits of a diet rich in fruits and vegetables, and the role of physical activity in GC prevention. Integrating dietary risk assessment into standard gastroenterological care, alongside screening and treatment for *H. pylori* infection, represents a practical, cost-effective strategy for primary prevention at the population level. The unexpected loss of significance for smoking and fruits warrants further investigation in the Fujian population, as these factors have shown associations in other populations. Future prospective studies are needed to confirm these patterns and to clarify the temporal and potentially causal relationships between combined lifestyle patterns and GC development.

## Figures and Tables

**Table 1:** Baseline characteristics and distribution of Healthy Lifestyle Index (HLI) components among gastric cancer cases and controls (N = 672).

Variables	Total (N = 672)	Cases (N = 336)	Controls (N = 336)	*p* Value
Age, years, mean ± SD	55.31 ± 10.83	56.76 ± 10.34	53.86 ± 11.13	**<0.001**
Age groups, years				**<0.001**
≤55	320 (47.6)	132 (39.3)	188 (56.0)	
>55	352 (52.4)	204 (60.7)	148 (44.0)	
Sex				1.000
Male	380 (56.5)	190 (56.5)	190 (56.5)	
Female	292 (43.5)	146 (43.5)	146 (43.5)	
BMI, kg/m^2^				0.081
<24	416 (61.9)	219 (65.2)	197 (58.6)	
≥24	256 (38.1)	117 (34.8)	139 (41.4)	
Marital status				**0.011**
Married	611 (90.9)	315 (93.8)	296 (88.1)	
Single/Separated/Divorced/Widowed	61 (9.1)	21 (6.3)	40 (11.9)	
Education level				**0.047**
Primary school & below	276 (41.1)	143 (42.6)	133 (39.6)	
Secondary & high school	282 (42.0)	148 (44.0)	134 (39.9)	
College & above	114 (17.0)	45 (13.4)	69 (20.5)	
Occupation				**0.012**
Housewives/Retired/Unemployed	234 (34.8)	125 (37.2)	109 (32.4)	
Farmers/Manual workers	199 (29.6)	99 (29.5)	100 (29.8)	
Business owners/service providers	172 (25.6)	91 (27.1)	81 (24.1)	
Office professionals	67 (10.0)	21 (6.3)	46 (13.7)	
Average monthly household income, RMB				0.287
<6000	304 (45.2)	160 (47.6)	144 (42.9)	
6000–12,000	278 (41.4)	137 (40.8)	141 (42.0)	
>12,000	90 (13.4)	39 (11.6)	51 (15.2)	
Residence				**<0.001**
Urban	357 (53.1)	194 (57.7)	163 (48.5)	
Rural	148 (22.0)	118 (35.1)	30 (8.9)	
Missing	167 (24.9)	24 (7.1)	143 (42.6)	
Family cancer history				0.813
No	394 (58.6)	247 (73.5)	147 (43.8)	
Yes	130 (19.3)	83 (24.7)	47 (14.0)	
Unknown	148 (22.0)	6 (1.8)	142 (42.3)	
Alcohol consumption				0.916
Yes	107 (15.9)	53 (15.8)	54 (16.1)	
No	565 (84.1)	283 (84.2)	282 (83.9)	
Smoking				**0.019**
Yes	233 (34.7)	131 (39.0)	102 (30.4)	
No	439 (65.3)	205 (61.0)	234 (69.6)	
Sedentary behavior				**<0.001**
Low	271 (40.3)	147 (43.8)	124 (36.9)	
High	225 (33.5)	177 (52.7)	48 (14.3)	
Missing	176 (26.2)	12 (3.6)	164 (48.8)	
Fruit intake				0.928
Healthy (≥200 g/day)	161 (24.0)	81 (24.1)	80 (23.8)	
Unhealthy (<200 g/day)	511 (76.0)	255 (75.9)	256 (76.2)	
Red meat intake				0.693
Healthy (≤300 g/week)	263 (39.1)	134 (39.9)	129 (38.4)	
Unhealthy (>300 g/week)	409 (60.9)	202 (60.1)	207 (61.6)	
Vegetable intake ^a^				0.110
Healthy (≥300 g/day)	422 (62.8)	201 (59.8)	221 (65.8)	
Unhealthy (<300 g/day)	250 (37.2)	135 (40.2)	115 (34.2)	
Pickled & processed food intake ^a^				**<0.001**
Healthy (≤5 g/day)	530 (78.9)	232 (69.0)	298 (88.7)	
Unhealthy (>5 g/day)	142 (21.1)	104 (31.0)	38 (11.3)	
Tea drinking				**0.002**
Healthy (daily)	103 (15.3)	66 (19.6)	37 (11.0)	
Unhealthy (less than daily)	569 (84.7)	270 (80.4)	299 (89.0)	
Regular eating habits				**<0.001**
Healthy (regular meals)	395 (58.8)	243 (72.3)	152 (45.2)	
Unhealthy (irregular meals)	277 (41.2)	93 (27.7)	184 (54.8)	
Physical activity ^b^				**<0.001**
Healthy (≥150 min/week MVPA)	165 (24.6)	107 (31.8)	58 (17.3)	
Unhealthy (<150 min/week MVPA)	507 (75.4)	229 (68.2)	278 (82.7)	

Data presented as N (%) unless stated otherwise. Bold *p* values indicate statistical significance (*p* < 0.05). ^a^ Thresholds based on the Dietary Guidelines for Chinese Residents 2022. ^b^ Based on the Physical Activity Guidelines for Chinese Residents 2021; MVPA = moderate-to-vigorous physical activity. BMI = body mass index; RMB = Chinese Yuan Renminbi; SD = standard deviation.

**Table 2:** Logistic regression analysis for the association between demographic factors and individual behavioral risk factors and gastric cancer.

	Cases	Controls	Univariate Logistic Regression		Multivariable Logistic Regression *	
			OR * (95 CI *)	*p* Value	OR (95 CI)	*p* Value
Sex						
Male	190 (56.5)	190 (56.5)	Reference	1.000		
Female	146 (43.5)	146 (43.5)	1.00 (0.73–1.36)			
Age groups, years						
≤55	132 (39.3)	188 (56.0)	Reference		Reference	
>55	204 (60.7)	148 (44.0)	1.96 (1.44–2.67)	**<0.001**	3.06 (1.91–4.89)	**<0.001**
BMI * (kg/m^2^)						
<24	219 (65.2)	197 (58.6)	Reference			
≥24	117 (34.8)	139 (41.4)	0.76 (0.55–1.04)	0.081		
Marital status						
Married	315 (93.8)	296 (88.1)	Reference		Reference	
Single/Separated/ Divorced/Widowed	21 (6.3)	40 (11.9)	0.49 (0.28–0.86)	**0.** **012**	0.45 (0.22–0.91)	**0.** **027**
Education						
Primary & below	143 (42.6)	133 (39.6)	Reference		Reference	
Secondary & High school	148 (44.0)	134 (39.9)	1.03 (0.74–1.43)	0.874	0.89 (0.54–1.47)	0.651
College & above	45 (13.4)	69 (20.5)	0.61 (0.39–0.95)	**0.** **027**	0.79 (0.38–1.65)	0.537
Occupation						
Housewives/Retired/ Unemployed	125 (37.2)	109 (32.4)	Reference		Reference	
Farmers/Manual workers	99 (29.5)	100 (29.8)	0.86 (0.59–1.26)	0.446	0.32 (0.17–0.60)	**<** **0.** **001**
Business owners/service providers	91 (27.1)	81 (24.1)	0.98 (0.66–1.45)	0.919	1.13 (0.62–2.07)	0.687
Office professionals	21 (6.3)	46 (13.7)	0.40 (0.22–0.71)	**0.** **002**	0.39 (0.17–0.88)	**0.** **023**
Average monthly household income (RMB)						
<6000	160 (47.6)	144 (42.9)	Reference			
6000–12,000	137 (40.8)	141 (42.0)	0.87 (0.63–1.21)	0.419		
>12,000	39 (11.6)	51 (15.2)	0.69 (0.43–1.11)	0.122		
Residence						
Urban	194 (57.7)	163 (48.5)	Reference	**<0.001**	Reference	
Rural	118 (35.1)	30 (8.9)	3.31 (2.10–5.19)		4.34 (2.42–7.78)	**<** **0.** **001**
Family cancer history						
No	247 (73.5)	147 (43.8)	Reference			
Yes	83 (24.7)	47 (14.0)	1.05 (0.70–1.59)	0.813		
Smoking						
Yes	131 (39.0)	102 (30.4)	Reference			
No	205 (61.0)	234 (69.6)	0.68 (0.50–0.94)	**0.** **019**	0.93 (0.58–1.48)	0.749
Alcohol consumption						
Yes	53 (15.8)	282 (83.9)	Reference			
No	283 (84.2)	54 (16.1)	1.02 (0.68–1.55)	0.916		
Sedentary behavior						
Low	147 (45.4)	124 (72.1)	Reference		Reference	
High	177 (54.6)	48 (27.9)	3.11 (2.09–4.63)	**<0.001**	4.06 (2.59–6.37)	**<0.001**
Fruit intake						
Healthy (≥200 g/day)	81 (24.1)	80 (23.8)	Reference			
Unhealthy (<200 g/day)	255 (75.9)	256 (76.2)	0.98 (0.69–1.40)	0.928		
Red meat intake						
Healthy (≤300 g/week)	134 (39.9)	129 (38.4)	Reference			
Unhealthy (>300 g/week)	202 (60.1)	207 (61.6)	0.94 (0.69–1.28)	0.685		
Vegetable intake						
Healthy (≥300 g/day)	201 (59.8)	221 (65.8)	Reference			
Unhealthy (<300 g/day)	135 (40.2)	115 (34.2)	1.30 (0.95–1.79)	0.103		
Pickled and processed food intake						
Healthy (≤5 g/day)	232 (69.0)	298 (88.7)	Reference		Reference	
Unhealthy (>5 g/day)	104 (31.0)	38 (11.3)	3.52 (2.33–5.29)	**<** **0.001**	1.75 (1.06–2.89)	**0.** **030**
Tea drinking						
Healthy (daily)	66 (19.6)	37 (11.0)	Reference		Reference	
Unhealthy (less than daily)	270 (80.4)	299 (89.0)	1.98 (1.25–3.05)	**0.** **002**	1.34 (0.82–2.20)	0.244
Regular eating habits						
Healthy (regular meals)	243 (72.3)	152 (45.2)	Reference		Reference	
Unhealthy (irregular meals)	93 (27.7)	184 (54.8)	3.08 (2.15–4.43)	**<** **0.** **001**	2.11 (1.47–3.03)	**<** **0.** **001**
Physical activity						
150 min/week MVPA	107 (31.8)	58 (17.3)	Reference		Reference	
<150 min/week MVPA	229 (68.2)	278 (82.7)	2.46 (1.68–3.59)	**<0.001**	1.98 (1.31–2.99)	**<** **0.** **001**

* Adjusted for age, marital status, education, occupation, average monthly household income, and residence. Data are presented as valid n (%) for categorical variables and mean ± SD for continuous variables. Bold *p* values indicate statistical significance (*p* < 0.05). BMI—Body Mass Index; RMB—Renminbi-Chinese Yuan; OR—Odds Ratio; CI—Confidence Interval.

**Table 3:** Distribution of the Healthy Lifestyle Index among the case and control groups.

Variable	Total (N = 672)	Cases (N = 336)	Controls (N = 336)	*p* Value
Healthy Lifestyle Index, mean ± std	4.81 ± 1.41	4.91 ± 1.47	4.71 ± 1.35	0.063
Healthy Lifestyle Index categories				0.078
Unhealthy	207 (30.8)	117 (34.8)	90 (26.8)	
Moderate	199 (29.6)	93 (27.7)	106 (31.5)	
Healthy	266 (39.6)	126 (37.5)	140 (41.7)	

**Table 4:** Logistic regression analysis for the association between the Healthy Lifestyle Index and gastric cancer.

Variables	Cases (N = 336)	Controls (N = 336)	Univariate OR (95% CI)	*p* Value	Adjusted OR (95% CI) *	*p* Value
Healthy Lifestyle Index						
Unhealthy (reference)	117 (34.8)	90 (26.8)	reference		reference	
Moderate	93 (27.7)	106 (31.5)	0.68 (0.46–1.00)	**0.049**	0.56 (0.31–1.00)	**0.049**
Healthy	126 (37.5)	140 (41.7)	0.69 (0.48–1.00)	**0.048**	0.34 (0.20–0.57)	**<0.001**

* Adjusted for age, sex, marital status, education level, occupation, average monthly household income, and residence. Bold *p* values indicate statistical significance (*p* < 0.05). OR = odds ratio; CI = confidence interval.

**Table 5:** Sensitivity analyses: continuous HLI and sex-stratified associations with gastric cancer risk.

Variables	Continuous HLI † AOR (95% CI)	*p* Value	Males AOR (95% CI)	*p* Value	Females AOR (95% CI)	*p* Value
Healthy Lifestyle Index						
Per 1-unit increase	1.34 (1.15–1.55)	**<0.001**	—	—	—	—
Unhealthy (reference)	—	—	reference		reference	
Moderate	—	—	0.29 (0.15–0.58)	**<0.001**	0.45 (0.17–1.15)	0.094
Healthy	—	—	0.35 (0.17–0.73)	**0.005**	1.32 (0.47–3.72)	0.604

All models adjusted for age, marital status, education level, occupation, average monthly household income, and residence. † Continuous HLI modeled as per 1-unit increase in score (range 0–10); higher scores indicate a greater number of unhealthy lifestyle behaviors. Bold *p* values indicate statistical significance (*p* < 0.05). AOR = adjusted odds ratio; CI = confidence interval.

## Data Availability

The data that support the findings of this study are available from the corresponding author, Yulan Lin, upon reasonable request. The data are not publicly available due to privacy and ethical restrictions.

## Supplementary Materials

The following supporting information can be downloaded at: https://www.mdpi.com/article/10.3390/nu18091343/s1, Table S1. Physical activity categories and MET scores adopted from The Adult Compendium of Physical Activities 2024. 1 MET = (3.5 mL O_2_ kg^−1^·min^−1^), Table S2. Comparison of healthy lifestyle index design, and the component classification of our study, Table S3. Spearman Rank Correlation Matrix of the Ten Healthy Lifestyle Index (HLI) Binary Components, Table S4. Variance Inflation Factor (VIF) Analysis for All Predictors in the Multivariable Model, Table S5. Multivariable Logistic Regression: Model Fit, Calibration, and Discrimination Statistics, Figure S1. Receiver Operating Characteristic (ROC) curve for the multivariable logistic regression model.

## Abbreviations

The following abbreviations are used in this manuscript:

GC	Gastric cancer
HLI	Healthy Lifestyle Index
BMI	Body Mass Index
CDG	Chinese Dietary Guidelines
RMB	Renminbi (Chinese Yuan)
MET	Metabolic Equivalent Task
MVPA	Moderate-to-Vigorous Physical Activity
CI	Confidence Interval
OR	Odds Ratio

## References

[1] Thrift A.P., El-Serag H.B. (2020). Burden of Gastric Cancer. Clin. Gastroenterol. Hepatol..

[2] Carcas L.P. (2014). Gastric Cancer Review. J. Carcinog..

[3] Yang L., Ying X., Liu S., Lyu G., Xu Z., Zhang X., Li H., Li Q., Wang N., Ji J. (2020). Gastric Cancer: Epidemiology, Risk Factors and Prevention Strategies. Chin. J. Cancer Res..

[4] Ferlay J., Mery L., Piñeros M., Znaor A., Soerjomataram I., Bray F., Laversanne M., Ervik M., Lam F., Colombet M. (2024). Global Cancer Observatory: Cancer Today.

[5] Thrift A.P., Wenker T.N., El-Serag H.B. (2023). Global Burden of Gastric Cancer: Epidemiological Trends, Risk Factors, Screening and Prevention. Nat. Rev. Clin. Oncol..

[6] He F., Wang S., Zheng R., Gu J., Zeng H., Sun K., Chen R., Li L., Han B., Li X. (2024). Trends of Gastric Cancer Burdens Attributable to Risk Factors in China from 2000 to 2050. Lancet Reg. Health West. Pac..

[7] Chen Y.-C., Fang W.-L., Wang R.-F., Liu C.-A., Yang M.-H., Lo S.-S., Wu C.-W., Li A.F.-Y., Shyr Y.-M., Huang K.-H. (2016). Clinicopathological Variation of Lauren Classification in Gastric Cancer. Pathol. Oncol. Res..

[8] Díaz del Arco C., Estrada Muñoz L., Ortega Medina L., Molina Roldán E., Cerón Nieto M.Á., García Gómez de las Heras S., Fernández Aceñero M.J. (2022). Clinicopathological Differences, Risk Factors and Prognostic Scores for Western Patients with Intestinal and Diffuse-Type Gastric Cancer. World J. Gastrointest. Oncol..

[9] Crew K.D., Neugut A.I. (2006). Epidemiology of Gastric Cancer. World J. Gastroenterol..

[10] Li L.F., Chan R.L.Y., Lu L., Shen J., Zhang L., Wu W.K.K., Wang L., Hu T., Li M.X., Cho C.H. (2014). Cigarette Smoking and Gastrointestinal Diseases: The Causal Relationship and Underlying Molecular Mechanisms (Review). Int. J. Mol. Med..

[11] Shah D., Bentrem D. (2022). Environmental and Genetic Risk Factors for Gastric Cancer. J. Surg. Oncol..

[12] Ma K., Baloch Z., He T.-T., Xia X. (2017). Alcohol Consumption and Gastric Cancer Risk: A Meta-Analysis. Med. Sci. Monit..

[13] Ma S., Liu H., Sun C., Meng M., Qu G., Jiang Y., Wu B., Gao J., Feng L., Xie P. (2023). Effect of Physical Activity on Incidence and Mortality in Patients with Gastric Cancer: Evidence from Real-World Studies. Cancer Causes Control.

[14] Maddineni G., Xie J.J., Brahmbhatt B., Mutha P. (2022). Diet and Carcinogenesis of Gastric Cancer. Curr. Opin. Gastroenterol..

[15] Berretta M., Cappellani A., Lleshi A., Di Vita M., Menzo E.L., Bearz A., Galvano F., Spina M., Malaguarnera M., Tirelli U. (2012). The Role of Diet in Gastric Cancer: Still an Open Question. Front. Biosci..

[16] Wang X.-Q., Terry P.D., Yan H. (2009). Review of Salt Consumption and Stomach Cancer Risk: Epidemiological and Biological Evidence. World J. Gastroenterol..

[17] Salvatori S., Marafini I., Laudisi F., Monteleone G., Stolfi C. (2023). Helicobacter Pylori and Gastric Cancer: Pathogenetic Mechanisms. Int. J. Mol. Sci..

[18] Bai X., Li X., Ding S., Dai D. (2023). Adherence to the Mediterranean Diet and Risk of Gastric Cancer: A Systematic Review and Meta-Analysis. Nutrients.

[19] Wang T., Cai H., Sasazuki S., Tsugane S., Zheng W., Cho E.R., Jee S.H., Michel A., Pawlita M., Xiang Y.-B. (2017). Fruit and Vegetable Consumption, *Helicobacter pylori* Antibodies, and Gastric Cancer Risk: A Pooled Analysis of Prospective Studies in China, Japan, and Korea. Int. J. Cancer.

[20] Yan X., Lei L., Li H., Cao M., Yang F., He S., Zhang S. (2023). Stomach Cancer Burden in China: Epidemiology and Prevention. Chin. J. Cancer Res..

[21] Zhang K., Yin J., Huang H., Wang L., Guo L., Shi J., Dai M. (2020). Expenditure and Financial Burden for Stomach Cancer Diagnosis and Treatment in China: A Multicenter Study. Front. Public Health.

[22] Mamun T.I., Younus S., Rahman M.H. (2024). Gastric Cancer—Epidemiology, Modifiable and Non-Modifiable Risk Factors, Challenges and Opportunities: An Updated Review. Cancer Treat. Res. Commun..

[23] Poorolajal J., Moradi L., Mohammadi Y., Cheraghi Z., Gohari-Ensaf F. (2020). Risk Factors for Stomach Cancer: A Systematic Review and Meta-Analysis. Epidemiol. Health.

[24] Liang J.L., Yuan H.M., Quan C., Chen J.Q. (2025). Risk Factors for Gastric Cancer: An Umbrella Review of Systematic Reviews and Meta-Analyses. Front. Oncol..

[25] Cheng L., Chen Y., Luo Z., Wang Q., Zou F., Lin Y. (2025). Evaluation of the Reliability and Validity of a Food Frequency Questionnaire Using Three-Day 24-Hour Dietary Recalls: A Study in Fujian, China. Nutrients.

[26] Herrmann S.D., Willis E.A., Ainsworth B.E., Barreira T.V., Hastert M., Kracht C.L., Schuna J.M., Cai Z., Quan M., Tudor-Locke C. (2024). 2024 Adult Compendium of Physical Activities: A Third Update of the Energy Costs of Human Activities. J. Sport Health Sci..

[27] Ding C., Feng G., Yuan F., Gong W., Yao Y., Ma Y., Zhang Y., Liu A. (2019). Temporal Trends and Recent Correlates in Sedentary Behaviors among Chinese Adults from 2002 to 2010–2012. Int. J. Environ. Res. Public Health.

[28] Foster H.M.E., Celis-Morales C.A., Nicholl B.I., Petermann-Rocha F., Pell J.P., Gill J.M.R., O’Donnell C.A., Mair F.S. (2018). The Effect of Socioeconomic Deprivation on the Association between an Extended Measurement of Unhealthy Lifestyle Factors and Health Outcomes: A Prospective Analysis of the UK Biobank Cohort. Lancet Public Health.

[29] Ding D., Rogers K., van der Ploeg H., Stamatakis E., Bauman A.E. (2015). Traditional and Emerging Lifestyle Risk Behaviors and All-Cause Mortality in Middle-Aged and Older Adults: Evidence from a Large Population-Based Australian Cohort. PLoS Med..

[30] Yuan Y.-Q., Li F., Dong R.-H., Chen J.-S., He G.-S., Li S.-G., Chen B. (2017). The Development of a Chinese Healthy Eating Index and Its Application in the General Population. Nutrients.

[31] China National Society (2022). Dietary Guidelines for Chinese Residents, 2022.

[32] Chinese Center for Disease Control and Prevention (2022). Eight Key Recommendations from Dietary Guidelines for Chinese Residents (2022).

[33] Zhao W.H., Li K.J., Wang Y.Y., Wang J., Liu A., Chen X., Xu J., Yang P., Ding C., Wang M. (2022). Physical Activity Guidelines for Chinese (2021). Chin. J. Public Health.

[34] He M., Lyu X. (2021). Application of BRAFO-Tiered Approach for Health Benefit-Risk Assessment of Dark Tea Consumption in China. Food Chem. Toxicol..

[35] Wu Y., Fan Y., Jiang Y., Wang Y., Liu H., Wei M. (2013). Analysis of Risk Factors Associated with Precancerous Lesion of Gastric Cancer in Patients from Eastern China: A Comparative Study. J. Cancer Res. Ther..

[36] IBM Corp (2019). IBM SPSS Statistics for Windows.

[37] Wang Z., Koh W.-P., Jin A., Wang R., Yuan J.-M. (2017). Composite Protective Lifestyle Factors and Risk of Developing Gastric Adenocarcinoma: The Singapore Chinese Health Study. Br. J. Cancer.

[38] Lynch B.M. (2010). Sedentary Behavior and Cancer: A Systematic Review of the Literature and Proposed Biological Mechanisms. Cancer Epidemiol. Biomark. Prev..

[39] Friedenreich C.M., Ryder-Burbidge C., McNeil J. (2021). Physical Activity, Obesity and Sedentary Behavior in Cancer Etiology: Epidemiologic Evidence and Biologic Mechanisms. Mol. Oncol..

[40] Maleki M., Fatehi V., Mohammadzadeh Z. (2024). The Association between Physical Activity and Risk of Gastric Cancer; an Umbrella Review. BMC Gastroenterol..

[41] Psaltopoulou T., Ntanasis-Stathopoulos I., Tzanninis I.-G., Kantzanou M., Georgiadou D., Sergentanis T.N. (2016). Physical Activity and Gastric Cancer Risk: A Systematic Review and Meta-Analysis. Clin. J. Sport Med..

[42] Ren J.-S., Kamangar F., Forman D., Islami F. (2012). Pickled Food and Risk of Gastric Cancer—A Systematic Review and Meta-Analysis of English and Chinese Literature. Cancer Epidemiol. Biomark. Prev..

[43] Yoo J.Y., Cho H.J., Moon S., Choi J., Lee S., Ahn C., Yoo K.-Y., Kim I., Ko K.-P., Lee J.E. (2020). Pickled Vegetable and Salted Fish Intake and the Risk of Gastric Cancer: Two Prospective Cohort Studies and a Meta-Analysis. Cancers.

[44] Feng X., Zhu J., Dai C., Hua Z., Zhu J. (2025). Coprevalence of Modifiable Gastric Cancer Risk Factors and Gastric Health among Chinese High-Risk Groups. Sci. Rep..

[45] Lim S.-L., Canavarro C., Zaw M.-H., Zhu F., Loke W.-C., Chan Y.-H., Yeoh K.-G. (2013). Irregular Meal Timing Is Associated with *Helicobacter pylori* Infection and Gastritis. Int. Sch. Res. Not..

