# Supplementary material for: The Combined Effect of Dietary and Behavioral Risk Factors in Gastric Cancer: A Case-Control Study Using a Healthy Lifestyle Index in Fujian, China
Source: Nutrients. 2026 Apr 24;18(9):1343. doi: 10.3390/nu18091343 (PMC13165403; doi:10.3390/nu18091343)
Supplement: Supplementary file 1 [file nutrients-18-01343-s001.zip › nutrients-4227494-supplementary.pdf]

Table S1. Physical activity categories and MET scores adopted from The Adult Compendium of Physical Activities 2024. 1 MET = (3.5 mL O2 · kg^{1} · min^{1}).

Category	Specific Activity	MET Score
**Occupational physical activity**	Predominantly sedentary (e.g., administrators, secretaries)	1.3
	Standing-oriented (e.g., salesperson, janitor)	1.8
	Predominantly general physical work (e.g., plumbing, carpentry)	3.3
	Predominantly heavy manual labor (e.g., loading, mining, steel making)	6.5
	Agricultural laborers (general, medium-intensity farm work in general/off-farm)	7.8
	Retired or unemployed/disabled	1.8
**Commuting physical activity**	Walking	4.0
	Riding a motorcycle	2.8
	Riding an electric bicycle	2.1
	Riding a regular bicycle	6.8
	Private/public transportation	1.3
	Usually work at home/or next to home	1.0
**Leisure physical activity**	Tai chi/qigong/walking	3.5
	Running/aerobics	7.3
	Ball games (basketball, table tennis, badminton, etc.)	7.5
	Brisk walking/health exercises/ranging	4.8
	Swimming	6.0
	Other (e.g., climbing)	5.0
**Domestic physical activity**	Household chores	4.0

MET-Metabolic Equivalent Task

Table S2. Comparison of healthy lifestyle index design, and the component classification of our study

Lifestyle Factor	Australian Cohort (Ding et al., 2015)	UK Biobank (Foster et al., 2018)	Fujian Study (Wangari et al., 2026)
Study Overview			
Design	Prospective cohort (45 and Up Study)	Prospective cohort (UK Biobank)	Hospital-based case-control
Setting	New South Wales, Australia	United Kingdom	Fujian Province, China
N	267,357	481,871 (328,594 NCD-free)	672 (336 cases, 336 controls)
Outcome	Mortality & CVD	Mortality & CVD	Gastric cancer risk
HLI Design			
Components (n)	5 broad domains	9 individual factors	10 individual factors
Scoring	Dietary index + binary factors	Binary: 0 = healthy, 1 = unhealthy	Binary: 0 = healthy, 1 = unhealthy
Score range	0–9	0–9	0–10
Categories	More healthy (0–3) Moderately healthy (4–6) Less healthy (7–9)	More healthy (0–3) Moderately healthy (4–6) Less healthy (7–9)	Tertiles: Healthy, Moderate, Unhealthy
Guideline basis	Australian Dietary Guidelines (2013)	Dietary Goals for Scotland (2016)	CDG 2022; PA Guidelines for Chinese (2021)
Component 1: Smoking			
Healthy	Past or never smoker	Past or never smoker	Past or never smoker
Unhealthy	Current smoker	Current smoker	>150 cigarettes/1 cigarette /day over 6 months
Component 2: Alcohol			
Healthy	≤14 drinks/week	≤4 times/week	No weekly consumption
Unhealthy	>14 drinks/week	Daily or almost daily	≥1 time/week
Component 3: Physical Activity			
Healthy	≥150 min/week MVPA (Active Australia Survey)	≥150 min/week moderate or ≥75 min/week vigorous (IPAQ)	Meets ≥150 min/week MVPA (all domains, ≥3·0 MET)
Unhealthy	<150 min/week	<150 min/week	Does not meet threshold
Component 4: Sedentary Behavior			
Healthy	<7 hours/day sitting	<4 hours/day TV viewing	<4 hours/day screen-based activities
Unhealthy	>7 hours/day sitting	≥4 hours/day TV viewing	≥4 hours/day screen-based activities
Component 5: Sleep Duration			
Healthy	7–9 hours/day	7–9 hours/day	Not included
Unhealthy	<7 or >9 hours	<7 or >9 hours	—
Rationale	Evidence for chronic disease risk	Mortality evidence	Weak gastric cancer-specific evidence
Component 6: Fruit Intake			
Healthy	≥2 serves/day (dietary index subscore; Australian guidelines)	Combined with vegetables: ≥400 g/day total	≥200 g/day (CDG 2022)

Lifestyle Factor	Australian Cohort (Ding et al., 2015)	UK Biobank (Foster et al., 2018)	Fujian Study (Wangari et al., 2026)
Unhealthy	<2 serves/day	<400 g/day combined	<200 g/day
Source	Australian Dietary Guidelines	UK Dietary Guidelines	CDG 2022; serving size from Chinese population norms
Component 7: Vegetable Intake			
Healthy	≥5 serves/day (dietary index subscore; Australian guidelines)	Combined with fruit: ≥400 g/day total	≥300 g/day (CDG 2022)
Unhealthy	<5 serves/day	<400 g/day combined	<300 g/day
Note	Combined in dietary index	Combined with fruit in one variable	Separate component
Component 8: Red Meat Intake			
Healthy	Not included in dietary score	≤3 portions/week	≤300 g/week (CDG 2022; ≤twice/week)
Unhealthy	—	>3 portions/week	>300 g/week
Items	Not assessed	Beef, lamb, pork (excl. processed)	Red meat + organ meats + chicken offal
Component 9: Processed/Pickled Meat & Food			
Healthy	<1 serve/week processed meat (dietary index subscore)	≤1 portion/week processed meat	≤5 g/day pickled & processed food (CDG 2022)
Unhealthy	>2 serves/week	>1 portion/week	>5 g/day
Items	Bacon, sausages, salami, burgers, etc.	Bacon, ham, sausages, pies, nuggets	Salted/century egg; pickled vegetables; fermented bean paste; processed pork/ham sausage
Rationale	Cancer prevention guidelines	Mortality evidence	Strongest dietary gastric cancer risk factor in Chinese population; salt-nitrite-H. pylori interaction
Component 10: Oily Fish/Seafood			
Classification	Dietary index subscore: <1 serve/week, 1–2, ≥2	≥1 portion/week oily fish	Not included as HLI component
Rationale	Included; protective in Australian diet	Included: mortality evidence	Excluded
Component 11: Tea Drinking (China-specific)			
Healthy	Not applicable	Not applicable	Daily tea consumption
Unhealthy	—	—	Less than daily
Rationale	Not relevant in Australian context	Not relevant in UK context	Antioxidant protection against H. pylori-related gastric cancer; culturally prevalent exposure
Component 12: Regular Meal Timing (China-specific)			
Healthy	Not captured	Not captured	Regular meal timing
Unhealthy	—	—	Irregular meal timing
Rationale	Not captured	Not captured	Linked to gastric acid dysregulation and favorable H. pylori environment; China-specific gastric cancer biological pathway
Components Not Included in Current Study			
Dairy	Milk type in dietary index (whole vs. skim)	Excluded; insufficient evidence for mortality	Excluded
BMI	Not included	Not included	Not included; BMI is partly an outcome of lifestyle, not a modifiable behavior

Lifestyle Factor	Australian Cohort (Ding et al., 2015)	UK Biobank (Foster et al., 2018)	Fujian Study (Wangari et al., 2026)
Sleep	Included	Included	Weak gastric cancer-specific evidence

Abbreviations: CDG, Chinese Dietary Guidelines for Residents 2022; CVD, cardiovascular disease; GC, gastric cancer; HLI, Healthy Lifestyle Index; IPAQ, International Physical Activity Questionnaire; MET, metabolic equivalent task; MVPA, moderate-to-vigorous physical activity; NCD, non-communicable disease; PA, physical activity.

Note: All HLI components in the current study scored 0 (healthy) or 1 (unhealthy). The 10-factor HLI score ranges from 0 (most healthy) to 10 (most unhealthy). Classification thresholds are derived from the CDG 2022 and the Physical Activity Guidelines for the Chinese Population (2021. Tea drinking and regular meal timing are novel China-specific components added on the basis of gastric cancer-specific biological plausibility in the Fujian study population.

Table S3. Spearman Rank Correlation Matrix of the Ten Healthy Lifestyle Index (HLI) Binary Components

Values are Spearman's ρ coefficients. Diagonal cells show "—" (self-correlation). Upper triangle is intentionally blank (matrix is symmetric). All variables are binary (0 = healthy, 1 = unhealthy).

HLI Component	1	2	3	4	5	6	7	8	9	10
1. Fruit intake	—									
2. Red meat intake	-0.093	—								
3. Vegetable intake	0.151	0.045	—							
4. Pickled and processed food intake	-0.102	-0.003	-0.082	—						
5. Smoking	0.160	-0.005	0.009	0.060	—					
6. Alcohol consumption	0.092	-0.001	0.010	0.054	0.409	—				
7. Physical activity	-0.097	-0.061	-0.090	0.050	-0.078	-0.054	—			
8. Sedentary behavior	0.003	0.059	-0.031	0.112	0.046	0.021	-0.093	—		
9. Tea drinking	0.321	0.023	0.020	-0.073	-0.280	-0.255	0.141	-0.101	—	
10. Regular meals	-0.093	-0.097	-0.013	-0.137	0.044	0.016	0.148	-0.229	0.113	—

Note. HLI = Healthy Lifestyle Index; ρ = Spearman rank correlation coefficient.

All pairwise correlations reflect statistical independence of HLI components assessed prior to multivariable modeling.

Table S4. Variance Inflation Factor (VIF) Analysis for All Predictors in the Multivariable Model

VIF was assessed via proxy linear regression incorporating all HLI binary components and sociodemographic covariates.
Values < 5 indicate acceptable collinearity; values < 10 indicate moderate collinearity.

Variable	VIF
**Sociodemographic covariates**	
Sex	1.121
Age	1.120
Education	3.723
Occupation	2.629
Average monthly family income	3.368
Marital status	1.266
Residence	1.223
**HLI binary components**	
Fruit intake	1.285
Red meat intake	1.357
Vegetable intake	1.120
Pickled and processed food	1.223
Smoking	1.121
Alcohol use	1.266
Physical activity	1.285
Sedentary behavior	1.120
Tea consumption	1.357
Regular meals (	1.223

Note. VIF = Variance Inflation Factor; HLI = Healthy Lifestyle Index.

VIF values were calculated using a proxy ordinary least squares regression model incorporating all predictors from the multivariable logistic regression model.

A VIF threshold of < 5.0 was applied to confirm the absence of problematic multicollinearity.

Table S5. Multivariable Logistic Regression: Model Fit, Calibration, and Discrimination Statistics

Results pertain to the fully adjusted model (outcome: Gastric Cancer Risk vs. controls; exposure: HLI percentile group with sociodemographic covariates).

Statistic	Value	df	p-value
**Omnibus test of model coefficients**			
Step	102.896	13	< 0.001
Block	102.896	13	< 0.001
Model	102.896	13	< 0.001
**Model summary**			
−2 Log likelihood	568.876	—	—
Cox & Snell R^2	0.184	—	—
Nagelkerke R^2	0.251	—	—
**Calibration**			
Hosmer–Lemeshow goodness-of-fit (χ^2)	5.595	8	0.692
**Discrimination**			
Area under the ROC curve (AUC)	0.754 (95% CI: 0.711–0.797)	—	< 0.001

Note. df = degrees of freedom; AUC = Area Under the Receiver Operating Characteristic Curve; ROC = Receiver Operating Characteristic; CI = Confidence Interval.

Omnibus test: $\chi^2(13) = 102.896$ indicates the model with all predictors fits significantly better than the null (intercept-only) model.

Hosmer–Lemeshow goodness-of-fit: $\chi^2(8) = 5.595$, $p = 0.692$ indicates adequate model calibration (non-significant χ^2 is desirable).

AUC = 0.754 (95% CI: 0.711–0.797, $p < 0.001$) reflects acceptable discriminative ability (AUC > 0.70 considered acceptable; > 0.80 excellent).

All statistics derived from SPSS v26 logistic regression output.

Figure S1. **Receiver Operating Characteristic (ROC) curve for the multivariable logistic regression model.**

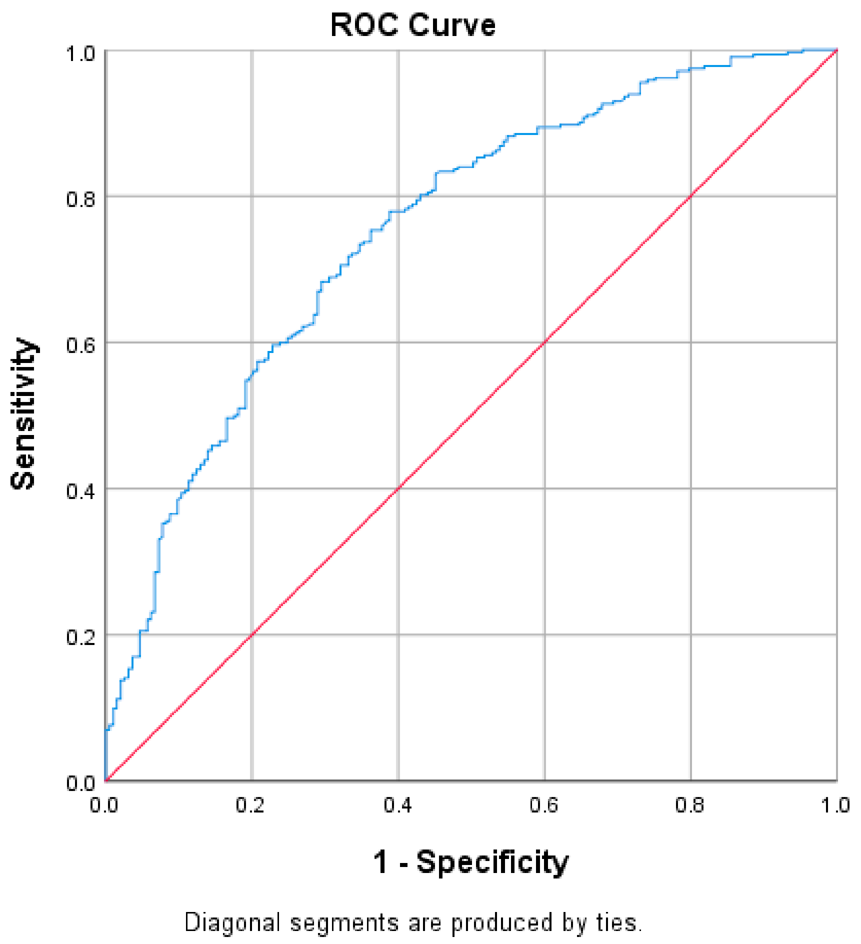

The blue line represents the sensitivity and specificity of the Healthy Lifestyle Index (HLI) and sociodemographic covariates in predicting gastric cancer risk.
The diagonal reference line indicates a model with no predictive power (AUC = 0.50).
The calculated Area Under the Curve (AUC) is 0.754 (SE = 0.022; 95% CI: 0.711–0.797; $p < 0.001$), indicating acceptable discriminative ability of the model.
